# Ultrafast two-photon fluorescence imaging of cerebral blood circulation in the mouse brain in vivo

**DOI:** 10.1073/pnas.2117346119

**Published:** 2022-06-01

**Authors:** Guanghan Meng, Jian Zhong, Qinrong Zhang, Justin S. J. Wong, Jianglai Wu, Kevin K. Tsia, Na Ji

**Affiliations:** ^a^Department of Molecular and Cell Biology, University of California, Berkeley, CA 94720;; ^b^Department of Physics, University of California, Berkeley, CA 94720;; ^c^Department of Electrical and Electronic Engineering, The University of Hong Kong, Hong Kong, China;; ^d^Advanced Biomedical Instrumentation Centre, Hong Kong Science Park, Hong Kong, China;; ^e^Helen Wills Neuroscience Institute, University of California, Berkeley, CA 94720;; ^f^Molecular Biophysics and Integrated Bioimaging Division, Lawrence Berkeley National Laboratory, Berkeley, CA 94720

**Keywords:** two-photon fluorescence, vasculature, hemodynamics, in vivo imaging, mouse brain

## Abstract

Characterizing blood flow dynamics in vivo is critical to understanding the function of the vascular network under physiological and pathological conditions. Existing methods for hemodynamic imaging have insufficient spatial and temporal resolution to monitor blood flow at the cellular level in large blood vessels. By using an ultrafast line-scanning module based on free-space angular chirped enhanced delay, we achieved two-photon fluorescence imaging of cortical blood flow at 1,000 two-dimensional (2D) frames and 1,000,000 one-dimensional line scans per second in the awake mouse. This orders-of-magnitude increase in temporal resolution allowed us to measure cerebral blood flow at up to 49 mm/s and observe pulsatile blood flow at harmonics of heart rate. Directly visualizing red blood cell (RBC) flow through vessels down to >800 µm in depth, we characterized cortical layer–dependent flow velocity distributions of capillaries, obtained radial velocity profiles and kilohertz 2D velocity mapping of multifile blood flow, and performed RBC flux measurements from penetrating blood vessels.

## Significance

Characterizing blood flow by tracking individual red blood cells as they move through vessels is essential for understanding vascular function. With high spatial resolution, two-photon fluorescence microscopy is the method of choice for imaging blood flow at the cellular level. However, its application is limited to a low flow speed regimen in anesthetized animals by its slow focus scanning mechanism. Using an ultrafast scanning module, we demonstrated two-photon fluorescence imaging of blood flow at 1,000 two-dimensional frames and 1,000,000 one-dimensional line scans per second in the brains of awake mice. These ultrafast measurements enabled us to study hemodynamic and fluid mechanical regimens beyond the reach of conventional methods.

The brain is nourished with a rich vascular network through which blood flow delivers nutrients and removes metabolic waste. The dynamics of blood flow (hemodynamics) are closely coupled with neural function and local metabolism, with many diseases of the brain having associated pathology in its hemodynamic characteristics (1–3). Ideal methods for characterizing blood flow in vivo should have sufficient spatiotemporal resolution to resolve capillaries and track red blood cell (RBC) motion. They should also be able to probe flow dynamics from blood vessels deep within the mammalian brain regardless of their orientation and should be capable of dealing with sample motion–induced artifacts.

Despite its importance, many existing methods for hemodynamic imaging fail to meet all the above requirements. For example, functional MRI (4) probes hemodynamics throughout the brain but is lacking in spatiotemporal resolution. Ultrafast ultrasound imaging can achieve capillary resolution millimeters below the skull but lacks sensitivity toward the smallest capillaries and individual RBCs (5–7). Laser Doppler and laser speckle imaging (8) have high temporal resolution but poor spatial resolution. Photoacoustic microscopy (9) can measure hemodynamic responses from individual vessels but also lacks single-RBC sensitivity and is limited in maximal detectable flow speed. Techniques based on optical coherence tomography (10, 11) have high spatiotemporal resolution, but their complex signal dynamics make them qualitative rather than quantitative tools for hemodynamic imaging (12).

Considered the gold standard for hemodynamics imaging when it comes to spatial resolution, two-photon fluorescence microscope (2PFM) scans tightly focused laser pulses across fluorescently labeled samples and record the two-photon–excited fluorescence signal at each focal position (13). With subcellular spatial resolution, 2PFM can monitor RBC movement in capillaries millimeters into the opaque rodent brain (14, 15) and has been extensively applied to study cortical blood flow (16).

The temporal resolution of 2PFM depends on the focus-scanning speed. With two-dimensional (2D) scanning, conventional 2PFM using a resonant galvanometer can achieve tens of frames per second (17). To increase temporal resolution, one-dimensional (1D) line trajectory scans along the vessels at up to thousands of lines per second have been used (14, 16, 18, 19). However, 1D scanning does not track vessel morphology and is susceptible to motion-induced artifacts. Consequently, most previous 2PFM hemodynamic imaging was performed in anesthetized animals with little sample-induced motion (14, 16, 18, 20, 21).

Here, we report ultrafast two-photon fluorescence imaging of cortical blood flow at a 1,000-Hz 2D frame rate and 1,000,000-Hz 1D line-scanning rate in the awake mouse cortex by using an ultrafast all-optical scanning method based on free-space angular chirp enhanced delay (FACED) (22, 23). With an orders-of-magnitude increase in temporal resolution, our system successfully measured cerebral blood flow up to 49 mm/s and revealed ultrafast flow dynamics at harmonics of the heart rate from cortical arterioles. With kilohertz 2D imaging and post hoc motion correction, we concurrently visualized RBC flow and vessel morphology at >800 µm in depth. The kilohertz frame rate also enabled us to characterize radial flow velocity profiles and achieve kilohertz 2D velocity mapping from venules and arterioles as well as RBC flux measurements from penetrating blood vessels.

## Results

### FACED 2PFM for Ultrafast Hemodynamic Imaging.

**Fig. 1.**
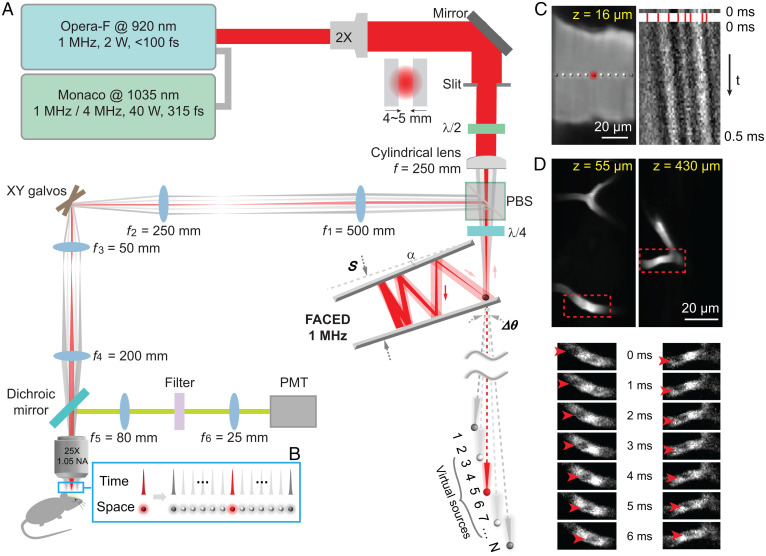
Ultrafast hemodynamic imaging in the awake mouse using a 2PFM with a FACED module. (*A* and *B*) A cylindrical lens, a polarizing beam splitter (PBS), a quarter-wave plate (λ/4), and a pair of nearly parallel mirrors form a FACED module that splits a laser pulse into multiple subpulses entering the microscope objective at distinct angles (e.g., red and gray beamlets) and time delays (*A*), forming a 1D array of spatially separated and temporally delayed excitation foci at the focal plane of a microscope objective (*B*). (*C*) *Left*, scanning the 1D array of FACED foci along a dural blood vessel. *Top Right*, a line scan is obtained every 1 μs. *Bottom Right*, stacking the line scans in time generates a kymograph, with RBCs appearing as dark streaks over a bright background of fluorescently labeled blood plasma. (D) *Top*, 2D images of blood vessels (average intensity projections of 5,000 2D images acquired at a 1-kHz frame rate). *Bottom*, zoomed-in view of individual frames imaged at a 1-ms interval. Red arrows indicate individual RBCs traveling through the blood vessels.

The principle and configuration of a FACED 2PFM was described in detail previously (23) (Fig. 1*A*). Briefly, a pulsed laser beam was focused in 1D with a convergence angle Δθ by a cylindrical lens, which was then directed into a pair of low-dispersion, high-reflectivity mirrors with separation S and a misalignment angle α. Beamlets entering the mirror pair at different angles then exited the mirror space after different numbers of reflections with distinct propagation directions. As a result, each laser pulse was split into multiple subpulses (N=Δθ/α) with 2S/c (c is the speed of light) interpulse temporal delay. Equivalently, the sequence of subpulses from the FACED module could be considered as light emanating from an array of virtual sources. Together, they formed a 1D array of spatially separated and temporally delayed excitation foci at the objective focal plane (Fig. 1*B*). With nanosecond time delay between the pulses, the fluorescence signal generated from distinct excitation foci could be temporally separated from the photomultiplier readout. Effectively, the FACED module achieved all-optical 1D line scanning at the repetition rate of the laser, which was 1 MHz in our case. Laser output at 920 nm or 1,035 nm was used for fluorescence excitation. We configured our FACED module to generate 80 to 100 foci at 1.0- to 2.0-ns temporal separation. By mechanically scanning the line of FACED foci perpendicular to the FACED focal array with a galvanometer (“galvo”), a 1-kHz 2D frame rate was achieved in an up to 80 µm × 140 µm imaging field of view (FOV). Detailed imaging parameters are summarized in *SI Appendix*, Table S1.

We demonstrated hemodynamic imaging in 1D at 1 MHz and 2D at 1 kHz (Fig. 1 *C* and *D*) in the cortex of head-restrained awake mice. Dextran-conjugated fluorescent dyes (fluorescein isothiocyanate [FITC] or Rhodamine B, *SI Appendix*, Table S1) were retro-orbitally injected to label the mouse blood plasma, with the unlabeled RBCs appearing as dark objects moving against a fluorescent background.

For 1D scanning, we oriented the brain so that the array of FACED foci scanned along the blood vessel. Keeping the galvos stationary, one 1D line scan was obtained every microsecond. Stacking line scans over time, we obtained a space–time image or a kymograph (Fig. 1*C*, from a 64-µm in diameter arteriole at 16 µm below the dura). RBCs traveling through the vessel lumen appeared as dark streaks whose angles with the time axis were determined by the flow velocity of RBCs (14, 24). The flow velocity was calculated from the kymograph using a cross-correlation–based particle imaging velocity (PIV) method (21), which we found to outperform methods based on Radon transform (14, 25–27) at large flow velocities (*SI Appendix*, *Supplementary Note*).

Scanning the 1D FACED focal array in the orthogonal direction with a galvo, we obtained full-frame images of cortical vessels at 1,000 frames/s. We were able to obtain vessel morphology while tracking the motion of individual RBCs from both superficial vessels and those at depth (Fig. 1*D*). These 2D time series were analyzed in two ways. For single-file flow, we extracted kymographs by manually drawing trajectories along the vessels and then used the PIV method to calculate blood flow velocity. For multifile flow vessels of larger diameters, we used the scale invariant feature transform (SIFT) flow method to map the flow velocity at 1 kHz and pixel resolution (28–30) (*Methods*). An algorithm that searches for matched pairs of pixels in two subsequent frames by solving a discrete optimization problem, SIFT flow allows for 2D velocity mapping with the pixel resolution at the imaging frame rate.

### Cerebral Hemodynamic Measurement at 1,000,000 Line Scans/s.

**Fig. 2.**
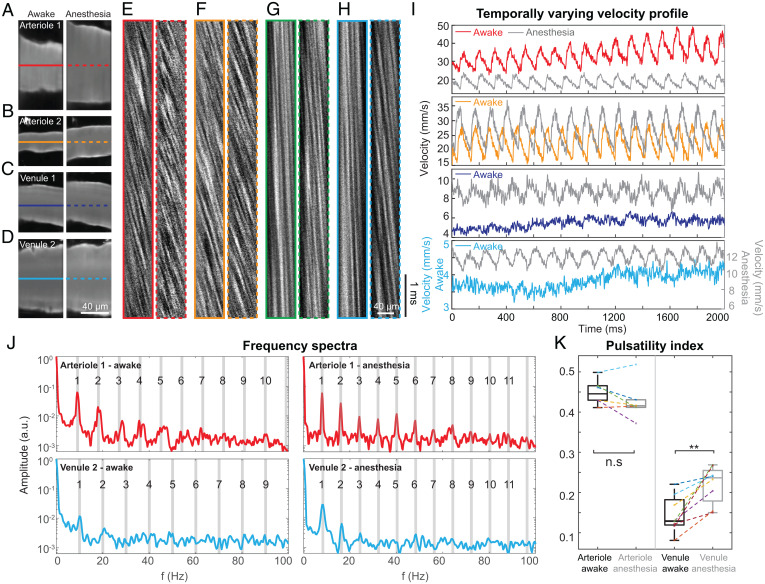
Line scanning at 1 MHz reveals ultrafast dynamics of high-speed flow in large blood vessels. (*A*–*D*) Images of blood vessels in awake (*Left*) or anesthetized (*Right*) mouse brains. Solid and dashed lines indicate trajectories of FACED megahertz line scanning in awake and anesthetized mice, respectively. (*E*–*H*) Kymographs generated at 1-µs temporal resolution for color-matched vessels in *A*–*D*. (*I*) Temporally varying velocity profiles calculated from kymographs in *E*–*H* using PIV method. (*J*) Frequency spectra of flow velocities measured from a representative arteriole (*A* and *E*) and venule (*D* and *H*); a.u., arbitrary units. Gray lines indicate fundamental and harmonic frequencies. (*K*) Comparison of pulsatility indices for arterioles and venules in awake or anesthetized mice. Paired *t* test; n.s., not significant; **, *P* < 0.01.

Taking advantage of its microsecond time resolution, we applied our megahertz line-scanning approach to measuring blood flow in superficial blood vessels of large diameters (up to 80 µm in diameter), where flow speed is expected to be high (Fig. 2). The same blood vessels were imaged under awake (Fig. 2 *A–D*, *Left*) and anesthetized states (Fig. 2 *A–D*, *Right*), and we observed vasodilation induced by isoflurane anesthesia, as reported previously (31). We then took 2-s–long line scans at 1 MHz and analyzed the resulting kymographs (Fig. 2 *E–H*) in which RBCs moving at higher speed generated more horizontal dark streaks (i.e., more vertical to the time axis).

At 1-MHz line scanning, we were able to capture blood flow with good contrast even in arterioles. As expected, the measured blood flow speed was much higher in arterioles than in venules. Whereas theoretically our line-scanning rate should enable the measurement of flow speed up to 25 m/s, the fastest flow that we observed experimentally was 49 mm/s (arteriole 1, Fig. 2 *A* and *I*).

We observed rapid variations in flow velocity, often in a pulsatile manner, in all the vessels examined (14 blood vessels from four mice). After Fourier transforming the temporally varying velocity profiles (Fig. 2*I*), we found that the pulsatile flow was, as expected, closely associated with the cardiac cycle (32) for both arterioles and venules (Fig. 2*J*). We observed up to eighth harmonic frequency bands of the fundamental heart rates from arterioles, with the widths of harmonic frequency bands becoming narrower under anesthesia for all arterioles. In contrast, no frequency bands beyond third harmonic were detected in any venule under either condition. This is consistent with previous arterial and venous pressure measurements, which reported a lack of higher harmonic oscillations in the pressure waveforms of veins (33).

Furthermore, whereas blood flow under anesthesia was found to be strongly pulsatile in both cortical arterioles and venules (Fig. 2*I*, gray traces) as in previous studies (20, 21), in the awake mice that we imaged, the pulsatility of blood flow was maintained in arterioles but suppressed in venules (Fig. 2*I*, colored traces). Pulsatility indices, defined as the difference between the peak systolic flow and minimum diastolic flow velocity divided by the mean velocity throughout the cardiac cycle (34), were calculated for the same vessels with and without anesthesia (six arterioles from two mice and eight venules from four mice; Fig. 2*K*). We found that arterioles overall have higher pulsatility than venules, and anesthesia did not have a significant impact on their pulsatility indices. In contrast, anesthesia significantly increased the pulsatility indices of venules (paired *t* test, *P* = 0.003).

### Kilohertz Full-Frame Recording Provides Simultaneous Measurement of Vessel Morphology and RBC Motion.

Despite its ultrahigh temporal resolution, megahertz FACED line scanning suffers the same limitation as other 1D scanning methods in that it does not provide information on vessel morphology. In addition, without imaging the vessels in 2D, sample motion during data acquisition cannot be corrected via post hoc image registration.

**Fig. 3.**
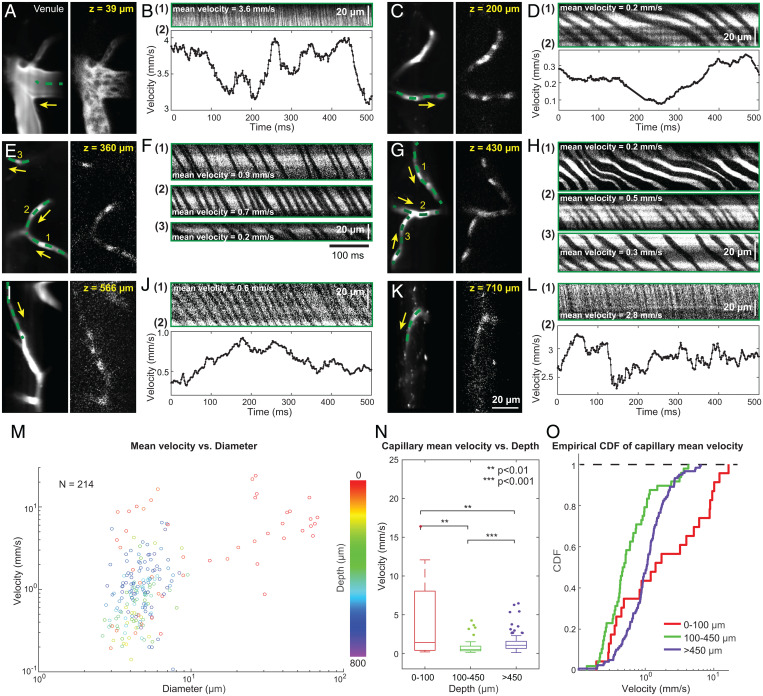
Flow velocity characterization throughout the mouse cortex with kilohertz full-frame two-photon imaging. (*A*, *C*, *E*, *G*, *I*, and *K*) *Left*, time-averaged images of example vessels. *Right*, single-frame images acquired at 1 kHz, resolving individual blood cells. Yellow arrows indicate flow direction. (*B*, *D*, *F*, *H*, *J*, and *L*) Example kymographs and velocity plots along the green dashed lines in *A*, *C*, *E*, *G*, *L*, and *K*. (*M*) Mean flow velocity versus blood vessel diameter color coded by vessel depth; *n* = 214 vessels. (*N* and *O*) Box and whisker plots (*N*) and empirical cumulative distribution functions (CDF) (*O*) of the mean flow velocity distributions for superficial, intermediate, and deep cortical capillaries; *n* = 190 capillaries. Maximal whisker length represents the interquartile range. Data were analyzed by Kolmogorov–Smirnov tests.

With FACED enabling kilohertz full-frame recording, we could record vessel morphology while measuring blood flow speed up to tens of millimeters per second (*Methods*), which covers the hemodynamics from all capillaries, most venules, and some arterioles in the mouse cortex (21). We performed kilohertz 2D imaging of vessels throughout the cortex of awake mice (Fig. 3). In individual 2D frames captured at 1 kHz (second columns, Fig. 3 *A*, *C*, *E*, *G*, *I*, and *K*), unlabeled RBCs surrounded by fluorescent plasma were clearly visualized. For large blood vessels with fast flow (Fig. 3*A*), individual RBCs traveling side by side were resolved at a 1-kHz frame rate but not in 100-Hz binned images (Movie S1), indicating the necessity of kilohertz imaging for capturing fast blood flow.

In capillaries, RBCs were resolved down to 820 µm below the dura surface (Fig. 3 *C*, *E*, *G*, *I*, and *K* and Movies S2–S5), where occasionally we observed cell aggregates and crescent-shaped cells (*SI Appendix*, Fig. S1*A*, white arrow and white arrowheads, and Movie S4). We manually selected pixels along the blood vessels and generated kymographs (Fig. 3 *B*, *D*, *F*, *H*, *J*, and *L*) to calculate flow velocity along these lines (green dashed lines, Fig. 3 *A*, *C*, *E*, *G*, *I*, and *K*). Kymographs of some capillaries had brightness variations along the line trajectories (e.g., horizontal dark stripes in Fig. 3 *D* and *H*), which were caused by slight vertical displacements of the capillaries relative to the focal plane. Given that these kymographs were generated from trajectories longer than 20 µm in length and the microscope’s excitation focus had an axial full width at half maximum of 2 to 4.5 µm, these displacements minimally impacted the accuracy of velocity calculation. Similar to large vessels, we observed substantial fluctuations in flow velocity for many capillaries (Fig. 3 *B*, *D*, *J*, and *L*). In some capillaries, blood flow even occasionally reversed direction (Movies S4 and S5), causing sign changes for the slopes in kymographs as well as the flow velocity derived with the PIV algorithm (*SI Appendix*, Fig. S1).

One important benefit of full-frame imaging is that we were able to track brain motion and eliminate its effect on velocity measurement. We found that, for our surgical preparations, superficial vessels did not need registration, likely because of their proximity to the cranial window used to stabilize the brain surface. However, capillaries as shallow as 200 µm in depth often had enough motion during 1-s–long recordings to introduce artifactual fluctuation and discontinuity to the velocity profile (*SI Appendix*, Fig. S2). Overall, we found that correction for brain motion via image registration (35) was essential for accurate velocity measurements in ∼80% of the vessels.

We measured the diameter and the mean flow velocity of 214 blood vessel segments within a depth range of 0 to 820 µm in five mice, including 190 capillaries with single-file flow and 3- to 10-µm diameters. We found a weak correlation between flow speed and blood vessel diameter (Fig. 3*M*; linear fitting, $R^{2}=0.24$). Separating the 190 capillaries into those in the superficial (0 to 100 µm, *n* = 23), intermediate (100 to 450 µm, *n* = 48), and deep (>450 µm, *n* = 119) layers, we found that although the diameter of capillaries in different layers had similar distributions (*SI Appendix*, Fig. S3), flow speed was highest in superficial capillaries, decreased in the intermediate layer, and increased again in deep vessels (Fig. 3 *M* and *N*). These differences in flow velocity were statistically significant (Kolmogorov–Smirnov test, Fig. 3*O*; *P* values for superficial versus intermediate, superficial versus deep, and intermediate versus deep capillaries of 0.003, 0.005, and 5.5 × 10^−6^, respectively, Fig. 3*N*), with median flow speeds of 1.42 mm/s, 0.48 mm/s, and 1.05 mm/s for superficial, intermediate, and deep vessels, respectively. This is consistent with previous anatomical mapping in the mouse showing that the cortical vascular network reached maximum branching around layer 4 (36), which may have led to the flow velocity drop in the intermediate cortical layer due to the increased flow capacity of its denser local vascular network (37).

### Kilohertz Full-Frame Imaging Measures RBC Flux and Velocity at Diverging Capillary Bifurcations.

Because full-frame images of multiple capillaries could be acquired at kilohertz with FACED 2PFM (Fig. 3 *E*–*H*), we characterized RBC flow at capillary bifurcations, an important fluid mechanical system (38) yet challenging for conventional 2PFM to study (39, 40).

**Fig. 4.**
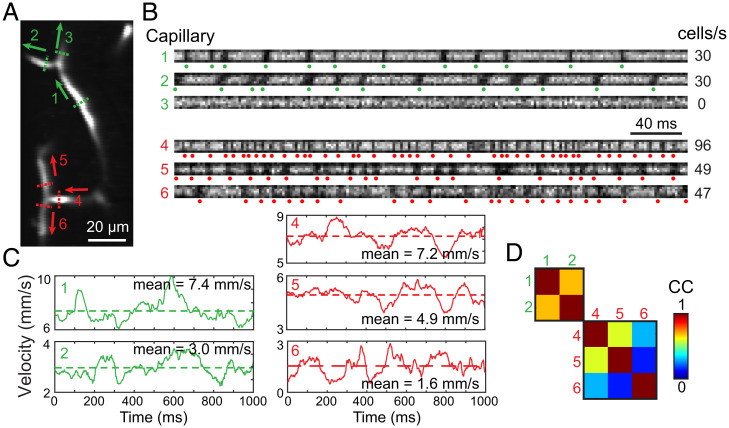
Kilohertz full-frame imaging enables quantification of RBC flux partition and flow velocity at capillary bifurcations. (*A*) Time-averaged image of two diverging capillary bifurcations; parent: 1; daughters: 2 and 3; parent: 4; daughters: 5 and 6. (*B*) Image representing the temporal variation of fluorescence signal across each capillary measured along dashed lines in *A*. Dots represent identified RBCs from images above. (*C*) Temporally varying velocity profiles of capillaries. No velocity was calculated for capillary 3 due to the absence of RBCs in the lumen. Dashed lines represent mean velocities. (*D*) Correlation coefficients (CC) between velocity profiles.

As a demonstration, we measured the RBC flux partition and flow velocity simultaneously in capillaries of two diverging bifurcations within the same FOV (26 µm below the dura; Fig. 4 and Movie S6). We generated an image representing the temporal variation of fluorescence signal across each capillary (along dashed lines, Fig. 4*A*) in which RBCs appeared as dark shadows (indicated by dots, Fig. 4*B*) and could be easily counted. RBC flux was then calculated as the number of RBCs passing through each capillary per second. In one bifurcation (parent: 1; daughters: 2 and 3; Fig. 4*B*), all RBCs from the parent capillary entered one of the daughter capillaries and caused the other channel to act as a plasma channel. In contrast, for the other bifurcation (parent: 4; daughters: 5 and 6; Fig. 4*B*), RBCs were evenly partitioned between two daughter capillaries (51% and 49% for capillaries 5 and 6, respectively). Because we performed RBC flux counting independently for each capillary, the fact that RBC fluxes of daughter capillaries added up to match the flux of the parent capillary for both bifurcations indicated the robustness of our measurements.

We also extracted kymographs along the capillaries and acquired their time varying profiles of RBC flow velocity (Fig. 4*C*). Interestingly, for these two bifurcations, RBC flux partition did not strongly correlate with flow velocity. Comparing the instantaneous flow velocities, we found that the daughter capillary that inherited all RBCs after the first bifurcation had only 42% ± 6% (mean ± SD) of the flow velocity of its parent capillary. For the second bifurcation, the flow velocity within one daughter capillary was 36% ± 14% of the other, yet they had similar RBC fluxes. Furthermore, whereas the flow velocities for parent capillary 1 and daughter capillary 2 were correlated in time, the flow velocity of parent capillary 4 was only correlated with daughter capillary 5 but not 6 (Fig. 4*D*). These results suggest that bifurcating capillaries do not always inherit the flow pattern from their upstream blood vessels, and, in contrast to general beliefs (38), the branch with a lower flow rate does not always receive disproportionally fewer RBCs.

### Kilohertz Full-Frame Hemodynamic Imaging Enables 2D Mapping of Blood Velocity Profiles within Vessels.

In large blood vessels with multifile flows, most previous two-photon hemodynamic imaging only measured the centerline flow velocity along the vessel axis (14, 16, 41, 42). To obtain a radial velocity profile from a cortical blood vessel, flow velocities at different radial positions had to be measured sequentially (20). With kilohertz full-frame recording through the central plane of blood vessels, we now can monitor multifile blood flow across the vessel lumen simultaneously and study its dynamics at high spatial and temporal resolution.

**Fig. 5.**
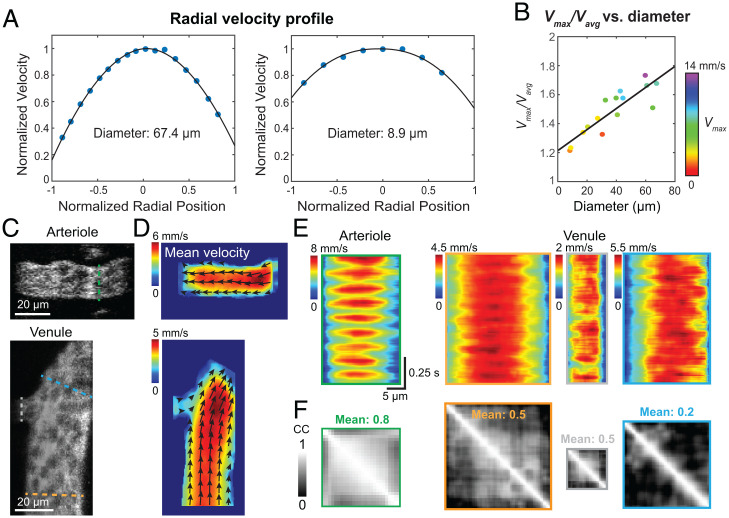
Kilohertz full-frame imaging enables in vivo fluid mechanics characterization. (*A*) Radial velocity profiles of two example vessels. Blue dots represent measured mean flow velocity over time. Black curves represent fits to blunted parabolic functions. (*B*) Velocity profile bluntness (*V*_max_/*V*_avg_) versus vessel diameter, with each vessel color coded by its *V*_max_. (*C* and *D*) Single frame images (*C*) and mean 2D velocity maps (*D*) for an unbranched arteriole and a converging venule branch, respectively. Arrows indicate flow direction (arrowheads) and velocity (arrow length). (E) Temporally varying radial velocity profiles extracted along dashed lines in *C*. (*F*) Pearson’s correlation coefficients (CC) between temporal velocity fluctuations at pixels along lines in *C*.

We first measured the radial velocity profiles of superficial cortical blood vessels in awake mice (15 vessels from four mice, 0 to 40 µm below the dura). We extracted kymographs by manually drawing trajectories along the vessel at different radial positions and determined the mean flow velocity for each radial position using 1D PIV analysis. The radial velocity profiles were generated with these mean velocities. We found the radial velocity profiles of these vessels to be blunter than the parabolic radial velocity profile of ideal laminar flow, resulting from the migration of RBCs away from the vessel wall during flow (38, 43). These velocity profiles were well fitted by a blunted parabolic function previously used to describe blood flow in the human retina (44) (average *R*^2^ of 0.97; Fig. 5*A* and *Methods*). We quantified the bluntness by the ratio *V*_max_/*V*_avg_ between the maximal flow speed *V*_max_ and the average flow speed *V*_avg_ along the profile, with smaller ratios corresponding to more blunted flow. We observed a positive correlation between *V*_max_/*V*_avg_ and vessel diameter (Fig. 5*B*, *R*^2^ = 0.76), which indicated that flow in smaller blood vessels had a blunter profile.

We applied the SIFT flow method to videos of multifile vessels in order to obtain their 2D velocity maps at 1 kHz and pixel resolution (*Methods* and Movies 7–11). Higher-velocity RBC cores can be easily identified from the 2D velocity maps, as exemplified by the mean velocity maps of an arteriole and a venule (Fig. 5*D*). With 1-kHz 2D flow mapping, we could extract radial velocity profiles at arbitrary vessel segments (dashed lines, Fig. 5*C*) and study their variation in time. We visualized the temporally varying radial velocity profiles by stacking them into 2D heat maps (Fig. 5*E*). The pulsatile flow of the arteriole was clearly revealed by the periodic oscillations of flow velocity in time (Fig. 5*E*, *Left*). Consistent with the 1-MHz line-scanning results discussed above, pulsatility of blood flow in the venules was more subdued (Fig. 5*E*, *Right*).

Furthermore, we calculated how the flow velocities at different radial positions correlated in time. In the unbranched arteriole, flows at different radial positions were highly correlated (mean Pearson’s correlation coefficients of 0.8; Fig. 5*F*, *Left*). In contrast, the convergence of flow for the venule branch caused disturbance to the flow in the output venule, as indicated by the lower correlation coefficients between its temporal velocity fluctuations at different radial positions (mean Pearson’s correlation coefficients of 0.5 for input venules and 0.2 for the output venule; Fig. 5*F*, *Right*).

### FACED 2PFM Enables RBC Flux Measurement in Penetrating Blood Vessels.

**Fig. 6.**
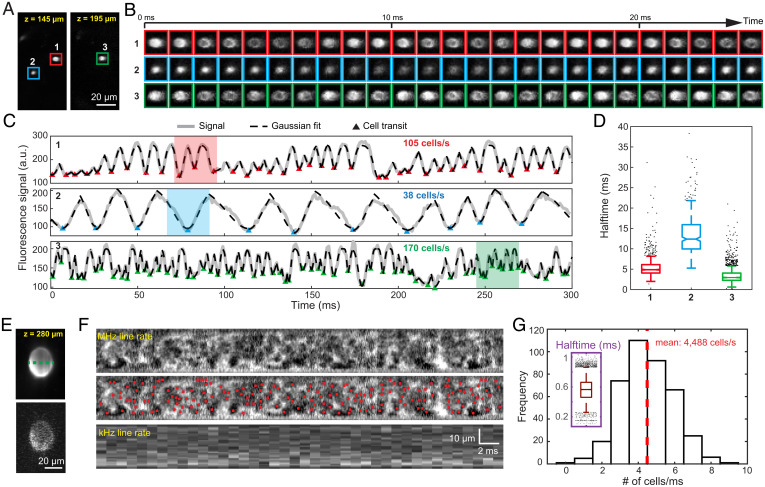
FACED 2PFM measures cell flux in penetrating blood vessels. (*A*) Time-averaged images of penetrating capillaries in an awake mouse brain. (*B*) Individual frames acquired at 1 kHz capturing RBCs (dark shadows) transiting through the excitation focus. (*C*) Averaged fluorescence signal traces of cross-sectional images of capillaries 1, 2, and 3 in *A*. Gray lines indicate signal. Dashed black lines indicate Gaussian fits. Triangles indicate an RBC transiting through focus. Shaded regions correspond to images in *B*. (*D*) The peak-to-valley or valley-to-peak half-times for RBCs detected in the three penetrating vessels. (*E*) Kilohertz 2D time-averaged (*Top*) and single frame (*Bottom*) of a large penetrating blood vessel under anesthesia. (F) Line scan along the green trajectory in *E* at 1 MHz (*Top* and *Middle*) and 1 kHz (*Bottom*). Red dots indicate identified RBCs transiting through focus. (*G*) Histogram of cell counts per millisecond. *Inset*, peak-to-valley or valley-to-peak half-times for RBCs detected in the large penetrating blood vessel.

Monitoring blood flow in vessels perpendicular to the imaging plane of 2PFM is a long-standing challenge (42) because these penetrating vessels show up in images as cross-sections (Fig. 6*A*), while extracting kymographs requires monitoring flow along the length of vessels. In principle, with the high axial resolution of 2PFM, RBCs traveling along such penetrating vessels can be detected from the transitory decreases in fluorescence of the vessel cross-sections caused by the unlabeled RBCs passing through the excitation focus. However, 2D imaging with standard 2PFM does not have sufficient temporal resolution to detect such transitory signal changes. One-dimensional line scanning has sufficient temporal resolution but cannot distinguish fluorescence signal changes caused by RBCs from sample motion artifacts.

With a 1-kHz frame rate, we successfully measured RBC flux from penetrating capillaries in the awake mouse brain. In consecutive frames acquired 1 ms apart (Fig. 6*B*), RBCs were visualized as dark shadows within the cross-sectional images of capillaries. Plotting the average brightness of the capillary cross-sections after image registration, we observed multiple local minima in the temporal traces of fluorescence (gray lines, Fig. 6*C*). A custom MATLAB code was used to count the number of local minima (triangles, Fig. 6*C*), which enabled us to calculate RBC flux within the capillaries. For these penetrating vessels, FACED 2PFM therefore functioned as an in vivo flow cytometer, counting the number of cells as they flowed through the imaging plane. Its high frame rate was essential for accurate cell counting in vessels with high flow; for a capillary with a flux of 170 cells/s (Fig. 6*C*, capillary 3), lowering the frame rate to 500 Hz (i.e., two-frame binning of the kilohertz data) missed 40% of cells, leading to an erroneous flux rate of 107 cells/s. Compared to flow velocity measurements on horizontal vessel segments, which we performed down to 820 µm in depth, a higher signal-to-noise ratio (SNR) was required to distinguish brightness changes due to blood cells from noise fluctuations. Nevertheless, we were able to measure cell flux from penetrating capillaries at beyond 400 µm in depth.

We measured the time that RBCs took to pass through the excitation focus. For each RBC detected, we fit the rising (RBC moving away from focal plane) and falling (RBC moving toward focal plane) edges of the fluorescence traces separately with Gaussian functions (dashed black lines in Fig. 6*C* and *Methods*) and found the average half-times for cells to travel through the focal plane to be around or less than 10 ms for the three capillaries (Fig. 6*D*).

Sometimes the kilohertz full-frame rate used above was not sufficiently fast to resolve individual transiting RBCs in high-velocity multifile flow through larger penetrating vessels (Fig. 6*E*). By acquiring line scans at 1 MHz through the center of the vessel cross-section (green dashed line, Fig. 6*E*), we quantified cell flux through one representative slice of the multifile flow, which gave rise to a lower bound of the total flux. To suppress motion artifacts, we acquired these 1-MHz line scans under anesthesia. The line scans acquired at different times formed a 2D image (Fig. 6*F*, *Top*), in which RBCs traveling through the focus appeared as dark clusters. By identifying the local minima (red dots, Fig. 6*F*) using an automatic pipeline (*Methods*), we calculated the cell flux along the scanning line every millisecond (Fig. 6*G*), which averaged 4,488 cells/s. The transmit time for this larger vessel was 0.6 ms (Fig. 6*G*, *Inset*), which was substantially smaller than those in the smaller capillaries due to its much faster flow. The kilohertz sampling rate was too slow to resolve RBCs flowing through this penetrating vessel (Fig. 6*F*, *Bottom*).

We can estimate RBC velocity in these penetrating vessels from the half-times. Using an RBC diameter of 6 µm (37) and the averaged half-time, the estimated flow velocities for the three single-file flow capillaries were 1.1 mm/s, 0.4 mm/s, and 2.5 mm/s, respectively, whereas the multifile flow had an estimated flow velocity of 12.5 mm/s. Note that RBCs tend to be stretched in high-speed flow (38); therefore, the estimated velocities were likely lower than the actual flow velocities, especially for multifile flow.

## Discussion

With the ultrafast line-scanning capability offered by a FACED module incorporated into a 2PFM, we demonstrated 1-MHz line scanning and 1-kHz full-frame recording of hemodynamics in the mouse cortex down to 820 µm in depth at cellular resolution in vivo. We applied FACED 2PFM to investigate a series of fluid dynamics questions related to blood flow.

Megahertz line scanning allowed us to measure fast blood flow in large vessels, with an observed maximal flow speed of 49 mm/s. Analyzing the temporal profile of flow speed, we found that, in addition to being strongly modulated at the heart rate, the pulsatile changes in flow speed also occurred at up to eighth harmonics of the heart rate in some arterioles. Similar harmonic oscillations in flow speed have been observed from carotid arteries by Doppler ultrasound (45) and MRI (46) but have not been measured at the level of individual cortical blood vessels. Pulsatility at harmonics of the cardiac cycle was also previously reported in human intracranial pressure measurements (47) and invasive cannula-based blood pressure measurements from arteries (33, 48). Our observations suggest that the pulsatility in blood pressure may be closely related to that in blood flow speed. Given that 8 to 10 harmonic orders were required to reconstruct a reasonable arterial blood pressure waveform (33, 48), the ability for FACED 2PFM to measure these high harmonics enabled us to accurately characterize the waveform of blood flow velocity as well. FACED 2PFM, therefore, is a valuable tool for investigating the interactions between pulsatile flow dynamics and vessel mechanical properties (e.g., oscillatory shear index [46, 49]) in individual vessels in vivo. Additionally, megahertz line scanning enabled RBC flux quantification of fast, multifile flow within a portion of penetrating vessel cross-section. The total RBC flux can then be estimated by adding up fluxes measured along multiple lines that tile the vessel cross-section or by assuming the same flux for multiple lines (e.g., an estimated total flux of 1.9 × 10^4^ cells/s for the vessel in Fig. 6*E*).

During circulation, RBCs are suspended in plasma and move through a geometrically complex network of vessels. Their mechanical interactions give rise to various hydrodynamic phenomena of blood flow, including the formation of a cell-depleted layer near the walls, the partition of RBCs at diverging bifurcations, and the blunted velocity profiles across the vessel lumen (38, 43), which can all be investigated by kilohertz full-frame imaging using FACED 2PFM. Cell-depleted layers were visualized in example videos acquired at a 1-kHz frame rate (Movies S7–S11). Therefore, our method can be used to characterize the temporal and spatial dynamics of the cell-depleted layers next to the vessel walls (50).

Although we did not systemically investigate RBC partition, our demonstration-of-principle experiment countered the belief that disproportionally more RBCs flow into the branch with a higher flow rate. Given the ability of FACED 2PFM to obtain vessel morphology, flow velocity, and cell flux simultaneously, it can be used for future investigations of how geometry and flow velocity of bifurcations influence RBC partition.

In order to understand the hydrodynamic interactions during blood flow, it is essential to measure velocity profiles of multifile flows within the vessel lumen. We observed blunted radial velocity profiles for multifile flows and found that the narrower the vessel (down to 8.3 µm in diameter), the more blunted its flow velocity profile was. Similar trends were observed previously in retinal blood vessels using a scanning laser ophthalmoscope through the human eye (44). However, due to the much lower spatial resolution of the ophthalmoscope, the previous study did not acquire data from blood vessels with diameters smaller than 30 µm. In contrast, we measured velocity profiles from vessels with diameters down to 8.3 µm and discovered a linear relationship between the bluntness and the vessel diameter. Combined with the pixel-resolution analysis of 2D flow velocities using SIFT flow, FACED 2PFM can be used to study how the spatial and temporal patterns of flow dynamics depend on parameters such as flow speeds as well as angles and diameters of diverging or converging branches.

The throughput of our system can be further increased by enlarging the FOV of the FACED 2PFM system through the optimization of the FACED module and increasing the recording length with a custom field-programmable gate array data acquisition and computing platform (51). Even faster line and full-frame scanning rates can be achieved by increasing the repetition rate of the excitation laser (e.g., 4-MHz line scanning and a 3-kHz 2D frame rate were achieved previously with a 4-MHz laser repetition rate [23]). Although the laser system used for 920 nm excitation of FITC is costly, the flexibility in the choice of fluorescent dyes for blood vessel labeling means that much cheaper, fixed-wavelength fiber lasers (e.g., the 1,035-nm output for the excitation of Rhodamine B) can be used for ultrafast hemodynamics imaging. Given its versatility in probing many aspects of hemodynamics, from flow velocity, vessel morphology, velocity profile, to cell counting in bifurcations and penetrating blood vessels, we expect FACED 2PFM to be widely applicable to and provide insights on hemodynamics in living organs.

## Methods

### A 2PFM with a FACED Module.

The simplified schematic of our microscope is shown in Fig. 1*A*, with the system setup described in detail previously (23). Detailed information on system alignment and configuration can be found in ref. 51. Briefly, 920-nm output was generated by an optical parametric amplifier (Opera-F, Coherent; 1-MHz repetition rate) pumped by a fiber laser at 1,035 nm (Monaco 1035-40-40, Coherent; 1-MHz repetition rate) for fluorescence imaging with FITC. The 1,035-nm output from the Monaco fiber laser was used directly to excite Rhodamine B. In both cases, the beam was expanded with a 2× beam expander (BE02M-B, Thorlabs), passed through a 4- to 5-mm–wide slit, and then focused one dimensionally (Δ*θ* = 1°) by a cylindrical lens (LJ1267RM-B, Thorlabs, effective input numerical aperture of 0.01) into a nearly parallel mirror pair (misalignment angle *α*, separation *S*, reflectivity of >99.9%, and group delay dispersion of <10 fs^2^ per reflection at 920 nm, fused silica substrate, 250 mm long and 20 mm wide; Layertec). The misalignment angle *α* and mirror separation *S* were adjusted to alter the number of foci in the FACED focal array and their temporal delay (for 2-ns, 1.5-ns, and 1-ns interpulse delays, *α* and *S* values were 0.0125° and 300 mm, 0.0188° and 225 mm, and 0.0125° and 150 mm, respectively). After the FACED module, the focal plane of the cylindrical lens (i.e., the location where the light entered and, after retroreflections, exited the mirror pairs) was conjugated to the midpoint of a pair of closely and orthogonally placed galvos (6215 H, Cambridge Technology), which were further conjugated to the back focal plane of a 25x/1.05-numerical aperture water-dipping objective lens (XLPLN25XWMP2, Olympus) by pairs of lenses. The two-photon excited fluorescence signal was collected by the same microscope objective, reflected by a dichroic mirror (FF665-Di02-25 × 36, Semrock), focused by two lenses (AC508-080-A and LA1951-A, Thorlabs), and, after passing through an emission filter (FF01-680/SP, Semrock), detected by a photomultiplier tube (H7422P-40 MOD, Hamamatsu). The photomultiplier tube signal was sampled at 625 MS s^−1^ (for 1.5-ns and 2-ns interpulse delay, PXIe-5160 embedded in PXIe-1071, National Instruments) or 10 GS s^−1^ (for 1-ns interpulse delay, ADQ7DC-PCIe, Teledyne SP Devices). Raw data from the digitizer were saved as 1D waveforms and then converted to tagged image format (TIF) images.

### Animal Preparation.

All animal experiments were conducted according to the National Institutes of Health guidelines for animal research. Procedures and protocols were approved by the Animal Care and Use Committee at the University of California, Berkeley. For this study, in vivo data were collected from nine wild-type mice (Jackson Laboratories, C57BL/6J). The cranial window implantation procedure has been described in detail previously (52). In brief, mice aged 3–4 mo were anesthetized with 1 to 2% isoflurane in O_2_, given the analgesic buprenorphine (subcutaneous [SC], 0.1 mg/kg), and head fixed in a stereotaxic apparatus (Kopf Instruments). A 3.5-mm–diameter craniotomy was made over the left V1 centered at −2.5 mm medial–lateral and 1 mm anterior–posterior to lambda. A glass window made of a single coverslip (Fisher Scientific, number 1.5) was embedded in the craniotomy and sealed by a tissue adhesive (VetBond, 3M). A stainless steel head bar was then firmly attached to the skull with dental acrylic. Implanted mice were provided with the postoperative analgesic meloxicam (SC, 5 mg/kg) for 2 d and allowed to recover for at least 2 wk before imaging experiments.

### In Vivo Imaging.

Unless specified, two-photon imaging experiments were performed on head-fixed, awake mice. As described previously (53), before imaging, animals were briefly anesthetized with isoflurane and retro-orbitally injected with 50 µL of 5% (wt/vol) dextran-conjugated fluorescent dyes with a molecular weight varying from 4 kDa to 2,000 kDa (53) (*SI Appendix*, Table S1). Mice were then head fixed under the objective lens. Imaging experiments started right after the mice woke up from anesthesia. Given the finite half-life of injected fluorescent dyes (∼2 h for 70-kDa dyes) and tissue attenuation of the excitation light, we first collected data from the deepest blood vessels and shifted the imaging plane gradually toward the brain surface.

For megahertz line scan, alignment of the blood vessel axis with the array of FACED foci was necessary to maximize the effective scanning FOV. A camera (Thorlabs Inc., DCC1545M-GL) was used to visually guide this process. FACED foci reflected by the glass cranial window and the superficial large-diameter blood vessels were both visible on the camera image. Using a manual rotation stage (RS65, Newport Inc.) mounted onto a three-dimensional translation stage (Dover motion, XYRB-1010), the lateral position and orientation of the mouse head was adjusted until the blood vessel axis coaligned with the FACED focal array.

### Choice of Fluorescence Dyes.

Ideal fluorescence dyes for FACED 2PFM should have a short fluorescence lifetime. Although FITC (∼4-ns lifetime) worked reasonably well when the FACED system was configured for 2-ns interpulse delay (50-µm–long 1-MHz line scan), we switched to Rhodamine B for the configuration with 1-ns interpulse delay (80-µm–long 1-MHz line scan) due to its ∼1.7-ns fluorescence lifetime.

### Data Processing and Analysis.

All image processing, analysis, and visualization, as detailed below, was performed in ImageJ (54) and MATLAB (MathWorks). All codes can be found at https://github.com/JiLabUCBerkeley/FACED2PFM-vessel.

### Intensity Normalization of FACED Pulses.

The pulse intensity within the array of FACED foci was not uniform, which led to varying signal brightness along each line scan. For better visualization, intensity normalization was performed as needed for most arterioles and venules with multifile flows (Figs. 1*C*, 2 *A–H*, and 5*C*), as detailed below, but not for capillaries. Except in Fig. 6*F*, where intensity normalization was required for accurate local minima identification and cell counting, it was not performed before any other quantitative analyses.

For 1D line scanning, the resulting kymographs were normalized using the 1D signal trace generated by summing the kymograph along the time dimension. For 2D full-frame imaging of multifile flow, we summed the pixel values along each horizontal line in the time-averaged image and sorted the horizontal lines by the summed signal. We then selected the 10 lines with the highest signal and summed them along the slow axis (perpendicular to the FACED foci). We then used the resulting 1D signal trace to normalize each line of the image. For both 1D and 2D cases, pixels close to the edge of the FOV that would have a >5× increase in signal by normalization were excluded from the normalization process and had their original value.

### Determination of the Maximum Measurable Flow Speed.

Because a kymograph requires at least two measurements on each cell in order to determine its flow speed, the cell displacement between consecutive measurements needs to be no more than half the length of FACED focal array. Therefore, the maximal measurable flow velocity is

$$v=f\cdot \frac{h}{2},$$

where h is the length of the FACED focal array, and f is the sampling rate. With a 50-µm–long FACED focal array, the maximal measurable flow speed was 25 mm/s at a 1-kHz 2D frame rate and 25 m/s at a 1-MHz line-scanning rate. With an 80-µm–long FACED focal array, the maximal measurable flow speed was 40 mm/s at a 1-kHz 2D frame rate and 40 m/s at a 1-MHz line-scanning rate.

### Kymograph Generation and Flow Speed Calculation.

 Kymographs were generated either directly from the 1-MHz line scans or from a resampling of the 1-kHz full-frame 2D images. For the full-frame data, a line-shape region of interest (ROI) covered pixels along the length of the blood vessel of interest in ImageJ. The ROI coordinates from ImageJ and the full-frame image stack were then loaded into MATLAB to generate the kymograph with custom code. To calculate flow speed, kymographs were fed into a correlation-based PIV code (21) (for a comparison with the Radon transform method, see *SI Appendix*, *Supplementary Note* and Fig. S4), along with parameters required for velocity calculation: frame rate and pixel size. Kymographs generated from 1-MHz line scans were temporally binned by a factor of 10 before being fed into the PIV pipeline. Kymographs from 1-kHz full-frame recording were either directly input into the PIV code or binned in time up to a factor of 5 to reduce analysis artifacts for data with low SNR (e.g., blood vessels at depth). The PIV code then extracted the flow velocity by estimating the cross-correlations between kymograph segments. The choice of the kymograph segments used for velocity analysis were user defined. For example, for data in Fig. 2*I*, after we calculated the average flow velocity of a 2-ms segment, another 2-ms–long segment that started 0.2 ms after the previous segment was used for velocity calculation. Repeating this process gave rise to 5 velocity measurement values per millisecond.

### Blood Vessel Diameter Calculation.

Blood vessel diameter was measured from the temporally averaged 2D image, where pixel brightness along a line orthogonal to the length of the blood vessel was first extracted. The line was determined as the orthogonal intersect of either the line ROI used to extract the kymograph ROI or the vessel edges detected by a Radon transform. The intensity profile along the line was then up-sampled by a factor of 10, after which the edges of the blood vessels were determined as the two positions with slopes of maximal amplitude and opposite signs. The diameter was calculated as the distance between these two edges.

### Flow Velocity Profile Fitting.

Adapted from a model proposed for human retinal blood vessels (44), the following equation was used to fit the radial flow velocity profiles from segments of venules and arterioles without nearby branch points or upstream to branch points

$$V\left(r\right)=V_{\text{max}}\;[1-\left(1-\beta \right)\;\cdot {|\frac{\left(r-r_{0}\right)}{R}|}^{B}],\;\left(0\leq |r|\leq R,\;0\leq \beta \leq 1\right),$$

where V(r) is the flow velocity profile as a function of radial position r, $V_{\text{max}}$ is the centerline velocity, R is the radius of the blood vessel, B is a bluntness index, and β is the extrapolated speed near the vessel wall. The asymmetry factor, $r_{0}$, was not in the previous model but was introduced here to account for any rotational asymmetry of the vessel cross-section and deviation between the blood vessel centerline and the imaging plane. We found that $r_{0}$ typically varied between 1% and 10% of the vessel radius. Parameters in Fig. 4 *A* and *B* were fitted using the mean velocities at each radial position.

To obtain the velocity bluntness, that is, *V*_max_/*V*_avg_, we adopted the equation from ref. 44

$$\frac{V_{\text{max}}}{V_{\text{avg}}}=\frac{B+2}{B+2\beta }$$

and inserted *B* and *β* obtained from fitting the blunted parabolic function. We then fit the velocity bluntness and vessel diameter in Fig. 5*B* with a linear function.

### SIFT Flow 2D Velocity Mapping.

The SIFT flow algorithm was described (28, 29) and its application to blood cell tracking reviewed (30) previously. Below, we outline two main steps in the SIFT flow method. The first step was feature extraction, which generated SIFT descriptors $s_{i}\left(p\right)$ that described the local gradient (i.e., orientation vector) at pixel ***p*** in frame *i*. In the case of blood vessel images, the most prominent features were the borders between the fluorescent plasma and the unlabeled RBCs. By using the pixel gradients rather than pixel values for further analysis, this step reduced the algorithm’s sensitivity toward motion-unrelated image variations (e.g., changes in global illumination or object rotation).

The flow field was then calculated from the extracted SIFT descriptors by solving a discrete optimization problem. The cost function was defined as in refs. 29 and 30,

$$\begin{array}{c} E\left(w\right)=\;\underset{p}{\sum_{}}\min\left({\left\|s_{i}\left(p\right)-s_{i+1}(p+w(p))\right\|}_{1},t\right)\\ +\underset{p}{\sum_{}}\eta \left(|u\left(p\right)|+|v\left(p\right)|\right)\\ +\underset{\left(p,q\right)\in \varepsilon }{\sum_{}}(\text{min}\left(\alpha |u\left(p\right)-u\left(q\right)|,d\right)+\text{min}\left(\alpha |v\left(p\right)-v\left(q\right)|,d\right)). \end{array}$$

Here, $s_{i}\left(p\right)$ was the SIFT descriptor extracted in the previous step, and w(p)=(u(p), v(p)) was the 2D flow vector at pixel location ***p***, with u(p) and v(p) being its horizontal and vertical components, respectively. w(p)  was obtained by minimizing the cost function, which included three terms. The first term (i.e., the data term) constrained the SIFT descriptors to minimally differ along the flow vector. The second term (i.e., the small displacement regularization term with weight *η*) constrained the flow vectors to be as small as possible. The third term (i.e., the smoothness regularization term with weight *α*) constrained the flow vectors of nearby pixels within the spatial neighborhood *ε* to be similar. *t* and *d* were truncation thresholds of *L*_1_ norms. In all the SIFT flow analysis in this paper, *η* was 0.01, *α* was 50, and *d* was 10,020 (*t* was the default in the SIFT flow demo script).

Since SIFT flow used a discrete optimization method, all images were up-sampled by a factor of 2 in pixel numbers via cubic interpolation before being processed by the pipeline to boost the precision of flow velocity calculation. For visualization of the flow field, a polygonal ROI was selected using ImageJ to define the edges of the vessel. The ROI was used as an input to the analysis pipeline to zero out the spurious flows outside the vessel footprint that were merely noise fluctuations before generating the final velocity maps. Note that the ROI was only used for visualization but not SIFT calculation.

As a validation, flow velocities calculated using the SIFT flow method were compared with those from 1D PIV (*SI Appendix*, Fig. S5). One-dimensional PIV analysis was performed on 61 manually drawn line ROIs from 12 blood vessels with multifile flows. The velocity values along the same lines were then extracted from SIFT flow outputs. Because SIFT flow generated flow vectors for all pixels along each line, which contained velocity information for both the faster moving RBCs and the slower moving blood plasma (38, 43), for each time point, we used the maximum flow velocity along the line trajectory to represent RBC velocity. The time-dependent velocity profiles extracted using these two methods closely resembled each other (*SI Appendix*, Fig. S5*A*) with similar time-averaged velocities (*SI Appendix*, Fig. S5*B*, linear fit, *R*^2^ = 0.91).

Generating 2D velocity maps at pixel resolution and imaging frame rate, the SIFT flow method had much higher spatial resolution than PIV methods. Because SIFT flow assumes that objects maintain their brightness and contrast while moving across the FOV, it worked well with large-diameter dural vessels that were within the focal plane (30). Because capillaries often deviated away from the objective focal plane within the FOV, the brightness and contrast variations of their RBCs and plasma (Fig. 3*G*) made SIFT flow a less reliable method than 1D PIV. Therefore, for flow analysis, we used 1D PIV for capillaries with single-file flow and SIFT flow for larger vessels with multifile flow.

### RBC Flux Measurement from Penetrating Blood Vessels.

To obtain RBC flux and flow speed from images of penetrating capillaries, an ROI enclosing the vessel cross-section was drawn in ImageJ. The ROI coordinates from ImageJ were then loaded into MATLAB with the kilohertz image stack to calculate the mean pixel brightness within the ROI. The intensity trace was smoothed by a Gaussian-weighted window with a width of three frames. It was then flipped upside down (valleys now became peaks) and processed by MATLAB function “findpeaks” to identify the moments when RBCs were within the excitation focus. Input parameters for the findpeaks function were adjusted for each dataset by visually comparing the identified cells in the temporal traces and the raw images. To find the half-time for RBCs to traverse the focal plane, the falling and rising edges associated with each identified cell were extracted and fitted separately using a Gaussian function with a negative amplitude. For each Gaussian fitting, the half-time was defined as the duration between 10% and 90% peak amplitude. The final half-time was calculated as the average half-time from both falling and rising edges.

For high-speed multifile flow through a large penetrating blood vessel, cell flux measurement was performed on a 2D image made of megahertz 1D line scans acquired at different times across the vessel cross-section. The image was first binned in time until the representations of individual RBCs appeared roughly round (100× binning for Fig. 6*C*) to facilitate the identification of local maxima using MATLAB function “ordfilt2”.

### RBC Flux Measurement at Capillary Bifurcations.

To measure RBC fluxes before and after capillary bifurcations, an image representing the temporal variation of fluorescence signal across each capillary was extracted from the kilohertz 2D frames along a line perpendicular to the capillary axis. The image was then summed along the space dimension, resulting in a 1D temporal signal. Steps afterward are the same as RBC flux measurement from penetrating capillaries described above; that is, the MATLAB function “findpeaks” was used to identify RBCs.

### Statistical Analysis.

Sample size, statistical tests, and *P* values are listed throughout the manuscript and figure captions.

## Supplementary Material

Supplementary File

Supplementary File

Supplementary File

Supplementary File

Supplementary File

Supplementary File

Supplementary File

Supplementary File

Supplementary File

Supplementary File

Supplementary File

Supplementary File

## Data Availability

TIF and binary MATLAB (MAT) files for all data in the manuscript have been deposited in the Dryad repository under “Ultrafast two-photon fluorescence imaging of cerebral blood circulation in the mouse brain in vivo” at https://doi.org/10.6078/D18M5V. All other data are included in the manuscript or supplementary information.
