# Supplementary material for: Ultrafast two-photon fluorescence imaging of cerebral blood circulation in the mouse brain in vivo
Source: Proc Natl Acad Sci U S A. 2022 Jun 1;119(23):e2117346119. doi: 10.1073/pnas.2117346119 (PMC9191662; doi:10.1073/pnas.2117346119)
Supplement: Supplementary File [file pnas.2117346119.sapp.pdf]

PNAS

Supplementary Information for
Ultrafast two-photon fluorescence imaging of cerebral blood circulation in the mouse brain in vivo

Guanghan Meng[a], Jian Zhong[b], Qinrong Zhang[b], Justin S. J. Wong[c], Jianglai Wu[b], Kevin K. Tsia[c,d,1], and Na Ji[a,b,e,f,1]

[a]Department of Molecular and Cell Biology, University of California, Berkeley, CA 94720

[b]Department of Physics, University of California, Berkeley, CA 94720

[c]Department of Electrical and Electronic Engineering, The University of Hong Kong, Hong Kong

[d]Advanced Biomedical Instrumentation Centre, Hong Kong Science Park, Hong Kong, China

[e]Helen Wills Neuroscience Institute, University of California, Berkeley, CA 94720

[f]Molecular Biophysics and Integrated Bioimaging Division, Lawrence Berkeley National Laboratory, Berkeley, CA 94720

[1]Corresponding authors: Kevin K. Tsia and Na Ji; Email: tsia@hku.hk and jina@berkeley.edu

This PDF file includes:

Supplementary Note
Figures S1 to S5
Table S1
Legends for Movies S1 to S11
SI References

Other supplementary materials for this manuscript include the following:

Movies S1 to S11

Supplementary Note

Comparison of PIV and Radon transform methods for kymograph analysis

We found that at high flow velocity, the PIV method was superior to an iterative Radon Transform method employed by us in a previous publication(1). To understand the origin behind this difference, we generated artificial blood flow data and tested the performance of these two methods against the ground truth (SI Appendix, Fig. S4).

We first generated the ground-truth temporally fluctuating positive velocity profiles by summing a constant baseline velocity with multiple sinusoidal functions with manually selected distinct amplitudes and frequencies. Next, we generated videos with 1,000 2D frames at 1 kHz containing a single blood vessel in which blood cells traveled at the ground-truth velocity. The positive flow direction was defined to be from left to right. Each frame was 50 µm × 50 µm, with a pixel size of 0.5 µm. For the first frame, pixels inside the vessel were initially set to the same user-chosen value while pixels outside the vessel was given the value 0. Then, we added 5 RBCs to the vessels that were almost equally distributed along the vessel but for random shifts of 1-5 pixels. The pixel values within the RBCs were modeled as a negative 2D Gaussian function with a standard deviation of 6 µm and a user-chosen amplitude. Gaussian noise was then added to the entire frame using MATLAB 'imnoise' function with a user-chosen SNR. Subsequent frames were generated the same way, except the locations of blood cells were calculated from those in the preceding frame using the ground-truth velocity profile. Once a blood cell exited the image from the right, a new blood cell was introduced from the left. Once the simulation video was generated, a kymograph was extracted along the centerline of the blood vessel. Radon transform and PIV analyses were then performed, and the extracted velocities compared with the ground truth (Fig. S4, images simulated with 5 sinusoidal functions for velocity profile, 128 for initial pixel value in vessel, amplitude of 60 for RBC image, 1dB for SNR). Radon transform mistook shadows of distinct RBCs as the streak left by a single RBC (Fig. S4 D, F), generating erroneous velocity measurements increasingly frequently at high flow speeds (Fig. S4 I, J), while PIV method reliably reproduced the ground truth up to 25 mm/s for 1 kHz 2D imaging data.

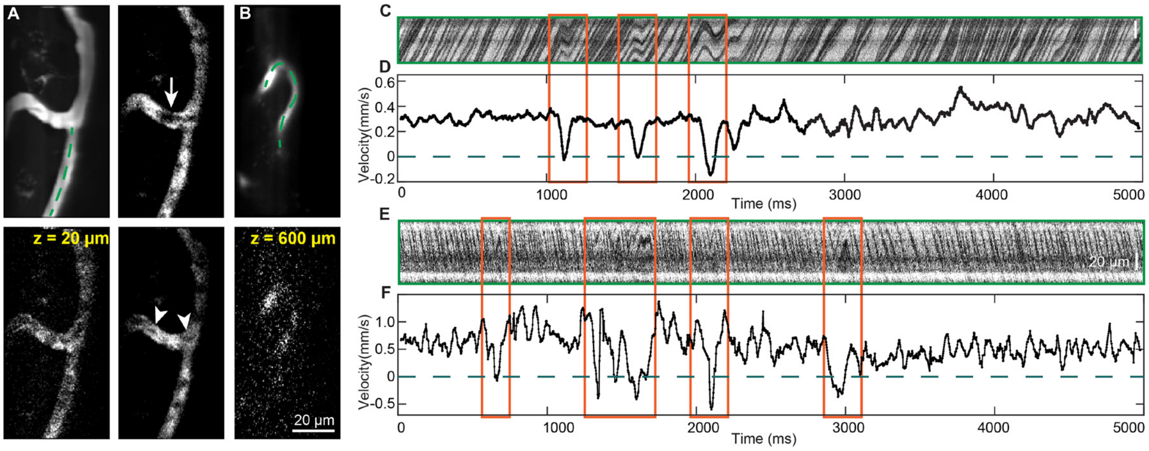

Figure S1. Cell morphology and flow reversion directly visualized by kHz full-frame imaging. (A) Superficial blood vessels with reversed flow. Top left: time-averaged image. Bottom left: example single-frame image. Top right: 4-frame average; White arrow: cell aggregate. Bottom right: 4-frame average: white arrowheads: crescent-shape cells. (B) (Top) Time-averaged and (Bottom) single-frame images of a deep capillary. (C, D) kymograph and velocity plot along the green dashed line in (A). (E, F) kymograph and velocity plot along the green dashed line in (B). Orange rectangles, periods containing reversed flows.

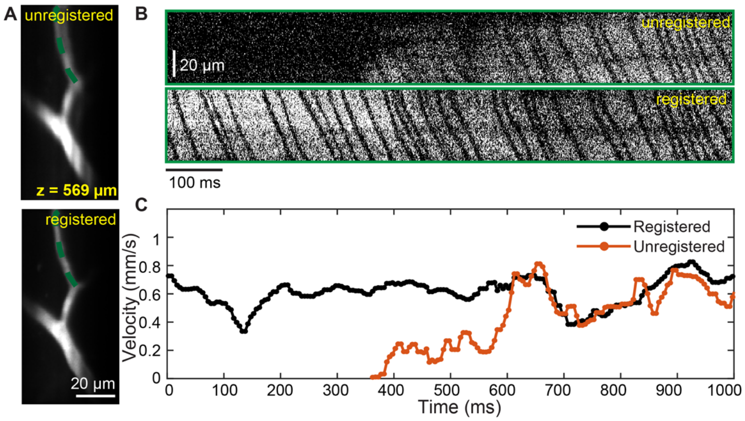

Figure S2. Image registration can be essential for accurate velocity measurement. (A) Unregistered (top) and registered (bottom) time-averaged images. (B) Kymographs extracted along the green dashed lines in unregistered and registered images, respectively. Note the missing section in the unregistered data due to vessel movement. (C) Flow velocity calculated from kymographs in (B). Note the velocity deviations between the unregistered and registered data, caused by motion artifacts.

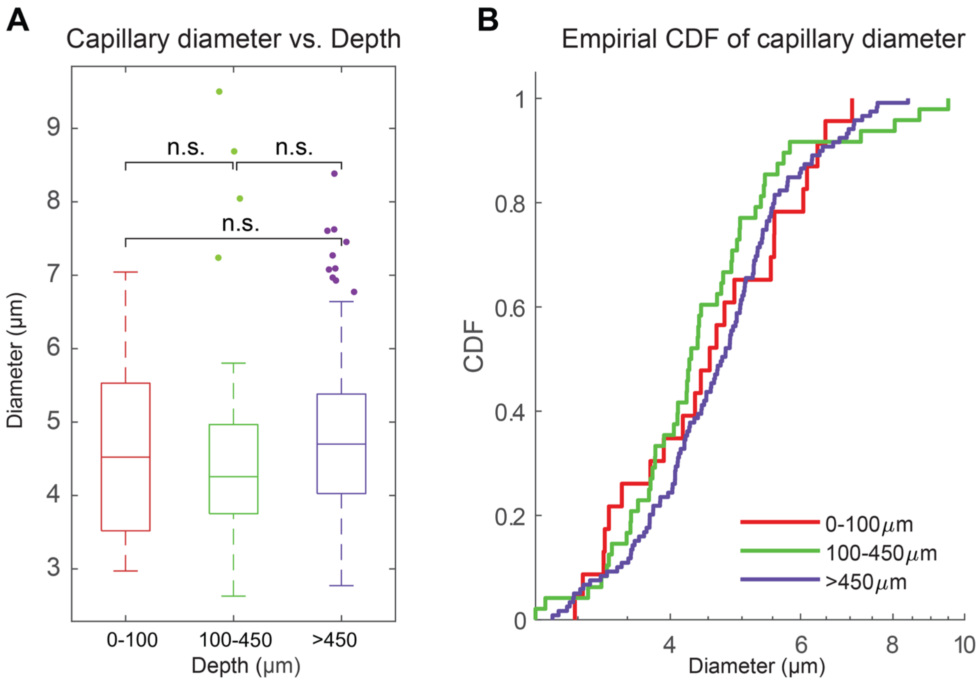

Figure S3. (A) Box-whisker plot and (B) empirical cumulative distribution functions (CDF) of capillary diameters for 23 superficial, 48 intermediate, and 119 deep cortical capillaries. Maximal whisker length: interquartile range. Statistical test: Kolmogorov–Smirnov tests. n.s.: not significant.

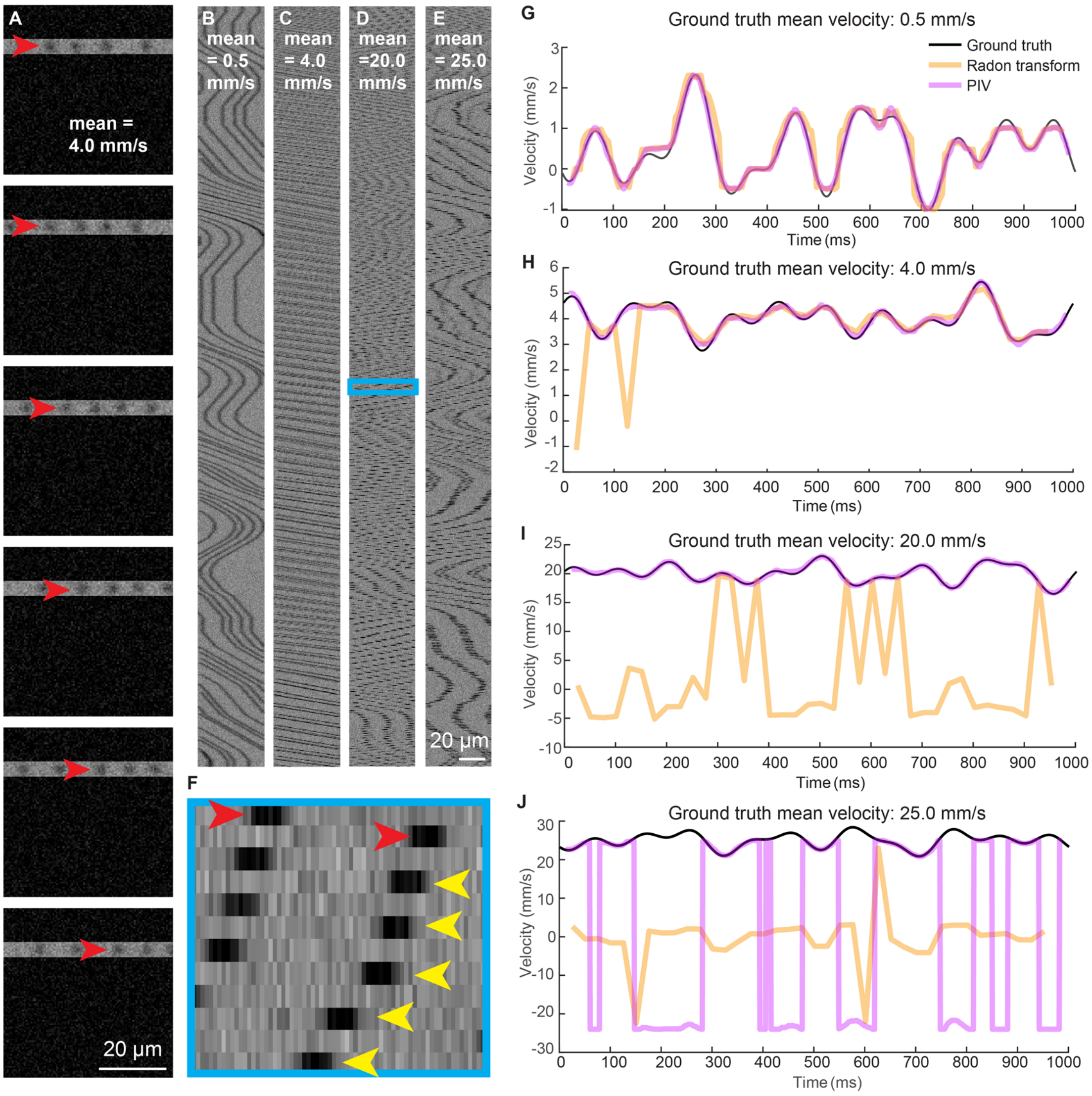

Figure S4. Cross-correlation-based PIV method outperforms iterative Radon transform in simulated blood flow images. (A) Individual frames of simulated 1kHz FACED images. Red arrowheads: a simulated blood cell traveling at an average speed of 4.0 mm/s. (B-E) Kymographs measured along the centerline of simulated vessel with average flow speed of 0.5, 4.0, 20.0, and 25.0 mm/s, respectively. (F) Zoomed-in view of the blue box in E. Red arrowheads: a fast-moving RBC. Yellow arrowheads: multiple distinct RBCs. (G–J) Extracted velocity profiles with PIV and Radon transform, compared with the ground truth.

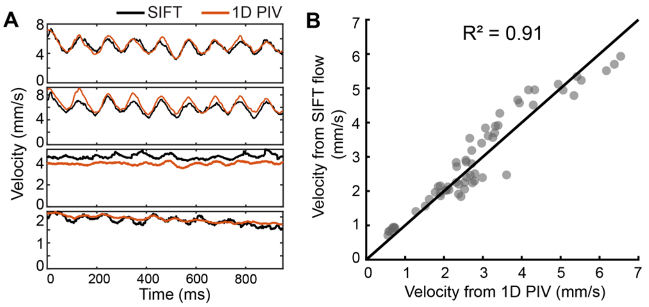

Figure S5. Comparison of results from SIFT flow and 1D PIV. (A) Example temporally varying flow profiles from SIFT-flow (black) and 1D PIV (red). (B) Correlation between the mean velocity from 61 line ROIs in 12 blood vessels with multi-file flows (gray dots) with the two methods. Black line has a slope of 1.

<table>
<tr><th>Figure number</th><th>Dye (Dextran conjugated)</th><th>Dye molecular weight</th><th>Imaging mode</th><th>FACED pulse separation (ns)</th><th>Laser wavelength (nm)</th><th>Laser</th><th>Laser power (mW)</th></tr>
<tr><td>Fig. 1C</td><td>Rhodamine B</td><td>70 kDa</td><td>MHz line scan</td><td>1</td><td>1035</td><td>Monaco</td><td>50</td></tr>
<tr><td>Fig. 1D</td><td>FITC</td><td>2 MDa</td><td>kHz full-frame</td><td>2</td><td>920</td><td>Opera-F</td><td>Left: 24
Right: 128</td></tr>
<tr><td>Fig. 2A,B,C</td><td rowspan="2">Rhodamine B</td><td rowspan="2">70 kDa</td><td rowspan="2">MHz line scan</td><td rowspan="2">1</td><td rowspan="2">1035</td><td rowspan="2">Monaco</td><td>50</td></tr>
<tr><td>Fig. 2D</td><td>90</td></tr>
<tr><td>Fig. 3A,B</td><td rowspan="6">FITC</td><td rowspan="5">2 MDa</td><td rowspan="10">kHz full-frame</td><td rowspan="5">2</td><td rowspan="6">920</td><td rowspan="6">Opera-F</td><td>24</td></tr>
<tr><td>Fig. 3C,D</td><td>81</td></tr>
<tr><td>Fig. 3E,F</td><td>115</td></tr>
<tr><td>Fig. 3G,H</td><td>128</td></tr>
<tr><td>Fig. 3I,J</td><td>128</td></tr>
<tr><td>Fig. 3K,L</td><td>4 kDa</td><td>1.5</td><td>96</td></tr>
<tr><td>Fig. 4</td><td rowspan="2">Rhodamine B</td><td rowspan="2">70 kDa</td><td rowspan="2">1</td><td rowspan="2">1035</td><td rowspan="2">Monaco</td><td>90</td></tr>
<tr><td>Fig. 5C, top</td><td>74</td></tr>
<tr><td>Fig. 5C, bottom</td><td rowspan="2">FITC</td><td rowspan="2">2 MDa</td><td rowspan="2">2</td><td rowspan="2">920</td><td rowspan="2">Opera-F</td><td rowspan="2">20</td></tr>
<tr><td>Fig. 6A</td></tr>
<tr><td>Fig. 6E</td><td>Rhodamine B</td><td>70 kDa</td><td>MHz line scan</td><td>1</td><td>1035</td><td>Monaco</td><td>107</td></tr>
<tr><td>Supplementary Fig. 1</td><td rowspan="2">FITC</td><td rowspan="2">2 MDa</td><td rowspan="2">kHz full-frame</td><td rowspan="2">2</td><td rowspan="2">920</td><td rowspan="2">Opera-F</td><td>128</td></tr>
<tr><td>Supplementary Fig. 2</td><td>10</td></tr>
</table>

Table S1. Sample and imaging parameters.

Movie S1 (separate file). 2D full-frame recording of fast blood flow in large pial blood vessels, within a 50 μm × 100 μm FOV. Individual RBCs travelling through the vessel lumen were resolved at 1 kHz frame rate (right panel) but not in the 100 Hz binned images (left panel).

Movie S2 (separate file). kHz 2D full-frame imaging of blood flow in capillaries 430 μm below dura surface (same data as in Fig. 3G). Flow dynamics from all visible blood vessel segments were measured simultaneously (Fig. 3H). FOV size: 50 μm × 100 μm.

Movie S3 (separate file). kHz 2D full-frame imaging of blood flow in capillaries at 430 μm below dura surface. The FOV (50 μm × 100 μm) was continuously shifted along the axis of FACED focal array to sequentially visualize capillaries in an area of 250 μm × 100 μm.

Movie S4 (separate file). kHz 2D full-frame imaging revealed reversed flow in capillaries at 20 μm below dura surface (same data as in Supplementary Fig. 1A, C, D). Red arrowheads: onsets of the reversal of blood flow direction. FOV size: 50 μm × 100 μm.

Movie S5 (separate file). kHz 2D full-frame imaging revealed reversed flow in a capillary at 600 μm below dura surface (same data as in Supplementary Fig. 1B, E, F). Red arrowheads: onsets of the reversal of blood flow direction. FOV size: 50 μm × 100 μm.

Movie S6 (separate file). kHz 2D full-frame imaging enabled monitoring of RBC velocity and flux partition from two capillary bifurcations at 26 μm below dura (same data as in Fig. 4). FOV size: 60 μm × 120 μm.

Movie S7 (separate file). Blood flow in an arteriole visualized by kHz full-frame imaging (same data as in Fig. 5C, D, top panels), with kHz 2D flow velocity mapping generated by SIFT flow analysis. Black arrows: flow speed (arrow length) and orientation. FOV size: 80 μm × 50 μm.

Movie S8 (separate file). Blood flow at a venous junction visualized by kHz full-frame imaging (same data as in Fig. 5C, D, bottom panels), with kHz flow velocity mapping generated by SIFT flow analysis. Black arrows: flow speed (arrow length) and orientation. FOV size: 50 μm × 100 μm.

Movie S9 (separate file). Blood flow at a venous junction visualized by kHz full-frame imaging (same data as in Fig. 3A,B, Supplementary Video 1), with kHz flow velocity mapping generated by SIFT flow analysis. Black arrows: flow speed (arrow length) and orientation. FOV size: 50 μm × 80 μm.

Movie S10 (separate file). Blood flow at a venous junction visualized by kHz full-frame imaging, with kHz flow velocity mapping generated by SIFT flow analysis. FOV size: 50 μm × 100 μm.

Movie S11 (separate file). Blood flow at a large venous junction visualized by kHz full-frame imaging, with kHz flow velocity mapping generated by SIFT flow analysis. Black arrows: flow speed (arrow length) and orientation. FOV size: 100 μm × 140 μm.

SI References

1. J. L. Fan *et al.*, High-speed volumetric two-photon fluorescence imaging of neurovascular dynamics. *Nat Commun* **11**, 6020 (2020).
